# Using plant physiological stable oxygen isotope models to counter food fraud

**DOI:** 10.1038/s41598-021-96722-9

**Published:** 2021-08-27

**Authors:** Florian Cueni, Daniel B. Nelson, Markus Boner, Ansgar Kahmen

**Affiliations:** 1 Department of Environmental Sciences – Botany, University of Basel, Schönbeinstrasse 6, 4056 Basel, Switzerland; 2 Agroisolab GmbH, Professor-Rehm-Strasse 6, 52428 Jülich, Germany

**Keywords:** Plant sciences, Plant physiology, Ecology, Agroecology, Biogeochemistry, Environmental economics, Stable isotope analysis

## Abstract

Fraudulent food products, especially regarding false claims of geographic origin, impose economic damages of $30–$40 billion per year. Stable isotope methods, using oxygen isotopes (δ^18^O) in particular, are the leading forensic tools for identifying these crimes. Plant physiological stable oxygen isotope models simulate how precipitation δ^18^O values and climatic variables shape the δ^18^O values of water and organic compounds in plants. These models have the potential to simplify, speed up, and improve conventional stable isotope applications and produce temporally resolved, accurate, and precise region-of-origin assignments for agricultural food products. However, the validation of these models and thus the best choice of model parameters and input variables have limited the application of the models for the origin identification of food. In our study we test model predictions against a unique 11-year European strawberry δ^18^O reference dataset to evaluate how choices of input variable sources and model parameterization impact the prediction skill of the model. Our results show that modifying leaf-based model parameters specifically for fruit and with product-independent, but growth time specific environmental input data, plant physiological isotope models offer a new and dynamic method that can accurately predict the geographic origin of a plant product and can advance the field of stable isotope analysis to counter food fraud.

## Introduction

Food fraud, the intentional and misleading adulteration of food for economic gain^1–3^, imposes an estimated annual burden of up to $40 billion^4–6^. Although most cases are not identified, it is estimated that 10% to 30% of all commercially sold food is fraudulent^4,5^. Improper labeling concerning the country of origin is the most common form of food fraud^7,8^, which is motivated by cost reduction and maximization of profits^1^. This erodes consumer trust^3^, and increases the potential for reduced quality and health risk^1,9^. Analytical tools for the independent verification of the geographical origin of food are therefore in high demand^10^.

The leading methods used for the forensic assessment of geographic food origin use stable isotope analyses^11–14^. Stable isotopes are especially ideal for verifying the origin of agricultural products, since climate and topography^15,16^, the underlying geology^17^, and agricultural practices^18^ lead to location specific isotopic fingerprints in a product^19^. Most methods using stable isotopes for the verification of geographical origins are based on the direct comparison of the isotopic fingerprint of a suspected sample to authentic reference material with known geographic origin. For such comparisons, statistical analyses are straightforward and data interpretation is easily understood by customers and law enforcement agencies^19^. Collecting authentic reference samples is, however, time consuming and expensive, especially on a global scale. Large reference datasets are therefore often geographically scattered and temporally not sufficiently resolved to account for the inter- and intra-annual variability observed in the oxygen and hydrogen isotope composition of plants, the two stable isotope ratios primarily used for origin analysis. This limitation can add substantial uncertainty in the provenance prediction of agricultural products.

In this study, we demonstrate how mechanistic plant physiological stable oxygen isotope models can be parametrized to serve as a fast, logistically simple, and low-cost alternative for predicting the geographic origin of agricultural plant products. The oxygen isotope composition (δ^18^O) of plant organic compounds is driven by the δ^18^O values of local precipitation^20–22^ and the evaporative environment of a plant, both of which show distinct geographic patterns^15,16^. If correctly parameterized, plant physiological stable isotope models can simulate how precipitation water δ^18^O values and climatic variables jointly shape the δ^18^O values of water inside the plant, and how these plant water δ^18^O values are imprinted into the plant’s organic materials^23,24^. Mechanistic plant physiological stable oxygen isotope models therefore have the potential to simulate the geographic variation of plant δ^18^O values (e.g. agricultural products), to which δ^18^O values of suspected food samples can be referenced and their purported geographic origin verified.

**Figure 1:**
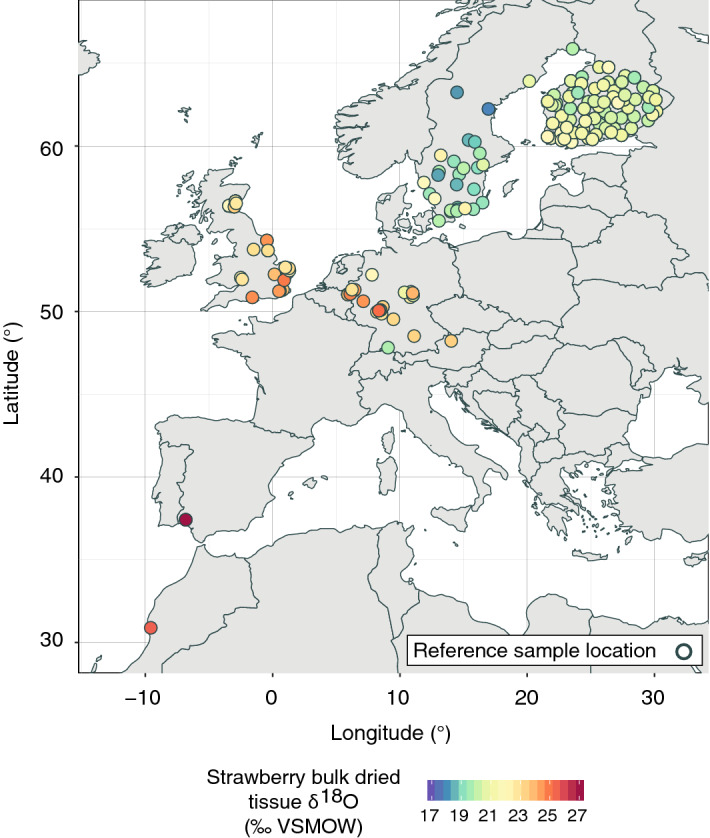
Map of Europe, showing the locations of the 154 authentic reference samples used for the model validation. The samples were collected between 2007 and 2017. The colored fill of the dots shows the measured δ^18^O values of the strawberry bulk dried tissue of the reference samples. The samples were mainly collected during the main European strawberry season from May to July^76^. The map was created using the software R, version 3.5.3 (https://www.r-project.org/).

To demonstrate conceptually how a mechanistic plant physiological stable isotope model can be used to simulate the geographic variability in δ^18^O values of food products, we use a unique Europe-wide strawberry δ^18^O reference dataset that contains 154 authentic reference samples that have been collected across Europe from 2007 to 2017 (Fig. 1). We employ this dataset to validate our model predictions across space and time and to test model assumptions that we make regarding model parameters and model input variables. With this approach we test if plant physiological stable oxygen isotope models that have originally been developed for leaf water or cellulose in the leaves or stems of plants are generally applicable for identifying the geographic origin of agricultural products, or if they need to be parameterized for specific plant species and their products. In addition, we carefully evaluate the type, necessary spatial resolution, and temporal integration of isotopic (precipitation δ^18^O values) and climatic (temperature, relative humidity) input variables that are required for the most accurate prediction of strawberry δ^18^O values across Europe.

## Results

**Figure 2:**
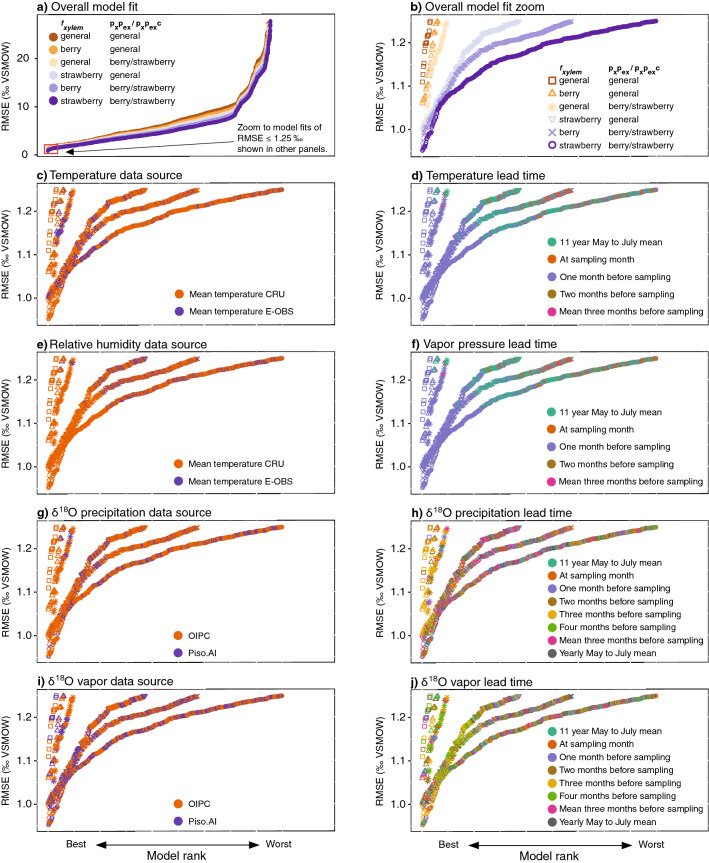
Model fit analysis of all possible parameter and model input data combinations. All panels show model root mean square error (RMSE) values obtained from comparison between modeled and measured bulk dried tissue δ^18^O values (n = 154) from authentic reference samples. Each dot represents the RMSE (in ‰ VSMOW) from one combination of model parameters and input data. RMSE values are ranked by best model fit. Panel (**a**) shows the ranked RMSE values that are lower than 25 ‰ from the total set of 65,536 tested combinations in each of the six differently parameterized models (n ≤ 14,850 for each parameter combination). The symbols are colored according to the different model parameter combinations (*f*_*xylem*_ and p_x_p_ex_/p_x_p_ex_c) used for the simulations, either general parameters for leaves averaged across various species using literature values (“general”), parameters that are average values for leaves (*f*_*xylem*_) and berries (p_x_p_ex_c) of berry producing plants (“berry”), or parameters specifically determined for leaves (*f*_*xylem*_) and berries (p_x_p_ex_c) of strawberry plants (“strawberry”). Panels (**b**–**j**) only show the models with RMSE values lower than 1.25 ‰ (red square panel **a**). Panel (**b**) shows the ranked model fits for the same model parameter and input data combinations as panel (**a**), but introduces symbols for the different model parameter combinations used. These symbols remain the same for panels (**c**–**j**). In panels (**c**–**j**) the symbols are colored based on the model input data used for the simulation (see panel in the figures for detail as well as Supplementary Information datasets S1 to S6). Panels (**c**) and (**e**) compare the use of CRU^29^ to E-OBS^30^ input data, panels (**g**), and (**i**) compare the use of OIPC^31^ to Piso.AI^32^ isotope data sources, and panels (**d**,**f**,**h**,**j**) compare the use of different lead time intervals leading up to the sampling date of the reference material (see methods). Since the panels only show the best model fits for RMSE values of up to 1.25 ‰, for panels (**d**) and (**f**) some time intervals yielded RMSE values greater than the plotted range, and are thus not indicated in the legends.

The model that we used in our simulations is based on the two-pool adapted Craig-Gordon model^25–27^. We followed a two-step approach to determine the best possible selection of model parameters and model input variables. Firstly, we utilized different combinations of the two key physiological model parameters. These are *f*_*xylem*_, which accounts for the dilution of ^18^O enriched water at the site of evaporation in leaves with the plant’s source water, and p_x_p_ex_/p_x_p_ex_c, which accounts for the extent of oxygen exchange between sugars and the surrounding plant water during biosynthesis of cellulose (p_x_p_ex_) and other compounds (p_x_p_ex_c). For these model parameters we used (i) mean values obtained from the literature that were originally determined for leaf water (*f*_*xylem*_) and leaf cellulose δ^18^O values (p_x_p_ex_) across various plant species. In addition we used values that were specifically determined for (ii) leaf water (*f*_*xylem*_) and bulk dried tissue of berries (p_x_p_ex_c) in berry producing plants and (iii) leaf water (*f*_*xylem*_) and bulk dried tissue of berries (p_x_p_ex_c) of strawberry plants in particular^28^ (see Table 1 and methods for a detailed description of the parameter selection). Since the p_x_p_ex_c for strawberries and berries of other berry producing plant species have been found to be identical^28^, this resulted in only one p_x_p_ex_c value together with one p_x_p_ex_ value, that we combined with three *f*_*xylem*_ values, and therefore six differently parameterized models (Fig. 2a). In a second step, we ran these six differently parameterized models with different combinations of environmental model input variables. This included different sources for precipitation δ^18^O data and climate data as well as different time periods over which to integrate these data leading up to the actual harvest date of a sample in the field (Table 2). The combination of different model input variables resulted in 65,536 different model simulations per model parameter combination (Fig. 2a). To evaluate the differences in performance among the different model parameter and input variable choices, we calculated root mean squared error (RMSE) values based on the comparison to our reference dataset for each of the 65,536 combinations of input variables for each of the six parameter combinations.

We found that all six differently parameterized models were able to predict the δ^18^O values of strawberry bulk dry material well. The best model performance was obtained using strawberry-specific model parameters, which yielded a minimum RMSE of 0.95 ‰. However, even the most general parameters still performed well, yielding a minimum RMSE of 1.11 ‰ (Fig. 2). Models that used average parameters (*f*_*xylem*_ and p_x_p_ex_c) for berry producing plants or parameters that are specific for strawberry plants, outperformed models that used at least one general parameter obtained from average literature values for leaves (Fig. 2a,b). The overall best model results were achieved using strawberry-specific model parameters (strawberry *f*_*xylem*_ and berry/strawberry p_x_p_ex_c) (Fig. 2a,b). The model that was parameterized with average parameters for berry producing plants (irrespective of the species) consistently showed the second-best performance (Fig. 2a,b). In particular, the choice of average berry producing plant or strawberry-specific values for the model parameter *f*_*xylem*_ over general across-species averaged literature values for *f*_*xylem*_ proved to be more important for increasing the model performance than the choice of p_x_p_ex_/p_x_p_ex_c values (Fig. 2a).

Our analysis further revealed that the choice of model input data strongly affects the quality of model output and that this is irrespective of the model parameterization (Fig. 2c–j). Out of the 65,536 different combinations of input parameter choices we tested, models using monthly mean temperature from the climatic research unit (CRU) (0.5° grid)^29^ performed better than models using data from the E-OBS dataset (0.1° grid)^30^, despite the lower spatial resolution of the CRU data (Fig. 2c). Similarly, using relatively humidity values derived from CRU vapor pressure data yielded better results than when using relative humidity data from E-OBS (Fig. 2e). The model comparisons also showed that the best predictions were obtained using temperature and vapor pressure input data for the month prior to the collection of a reference sample (Fig. 2d,f, Supplementary Information dataset S1 to S6). For precipitation δ^18^O data, the models using monthly values from the OIPC^31^ (which are not specific for a given year) generally showed a better fit (26 out of the 30 lowest RMSEs) than those using Piso.AI^32^, which predicts values for specific months and years (Fig. 2g). Still, the single best fit values were based on Piso.AI derived mean of the three-month period leading up to sampling (Supplementary Information dataset S6). For vapor δ^18^O values, however, the choice of data source showed no such clear pattern (Fig. 2i). In contrast to the temperature and vapor pressure data, the selection of the time interval prior to the collection of the reference sample resulted in no consistent best choice for precipitation and vapor δ^18^O input variables (Fig. 2h,j).

**Figure 3:**
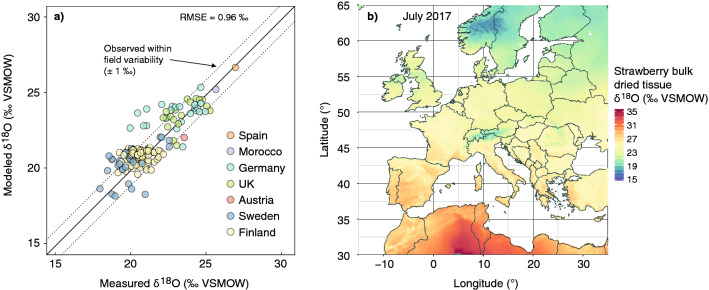
Results of the simulation of berry bulk dried δ^18^O values using plant physiological isotope models. (**a**) The modeled bulk dried tissue δ^18^O values obtained from the model that was parameterized with general data for berries plotted against measured authentic bulk dried tissue reference samples δ^18^O values (n = 154; RMSE = 0.96 ‰). Solid line represents the 1:1-line, the dashed lines show the 95% quantile of the observed within field variability of strawberry δ^18^O bulk dried tissue values. (**b**) Map of Europe showing the expected spatial distribution of bulk dried tissue δ^18^O values of strawberries, in this example collected in July 2017. The map was created using the software R, version 3.5.3 (https://www.r-project.org/).

Comparing all possible model parameter and input data choices, we found two nearly identically best performing models. These were the model that used average model input parameters for berry producing plants, which yielded an RMSE of 0.96 ‰, and the model that used strawberry-specific parameters, which yielded an RMSE of 0.95 ‰ (Figs. 2, 3). The best performing model using average parameters for berry producing plants used monthly air temperature and vapor pressure data from CRU from one month before the collection of a reference sample in a specific year, precipitation δ^18^O values of the precipitation-weighted mean of the three-month period leading up to the harvest date of a sample from the long term mean monthly values provided by OIPC, and vapor δ^18^O values calculated assuming isotopic equilibrium with the precipitation δ^18^O values three month before harvest.

The map of predicted strawberry bulk dried tissue δ^18^O values from this best performing average berry model for the example month of July 2017 shows values ranging from + 31 ‰ to + 16 ‰ across the European continent (Fig. 3b). The measured δ^18^O variability within one region was generally higher than the variability captured by the model, with the largest outliers sourcing from eight locations in Germany and Sweden, which the model over or underpredicted by 2.0 ‰ to 3.5 ‰ (Fig. 3a). However, the model induced uncertainty fell mostly within the 95% quantile of the observed within-field variability of strawberry bulk dried tissue δ^18^O values (± 1 ‰) which we obtained through berry samples that were collected on the same field and the same day (mainly from Germany and Finland) (Fig. 3a).

## Discussion

We demonstrate that a plant physiological stable oxygen isotope model that allows for the prediction of δ^18^O values of organic compounds in leaves and wood can also serve as a powerful tool for predicting the spatial variability of δ^18^O values in fruit. With the best choice of environmental input data, models parameterized with general across-species averaged leaf-derived values for the model parameters *f*_*xylem*_ and p_x_p_ex_ can predict observed data with an average RMSE of 1.11 ‰. The model that best predicted δ^18^O values of bulk dried tissue of strawberries required model parameters specifically defined for leaves (*f*_*xylem*_) and berries (p_x_p_ex_c) of strawberry plants. This model was, however, only marginally superior to the best performing model that was parameterized with average values for leaves (*f*_*xylem*_) and berries (p_x_p_ex_c) of berry producing plants in general (RMSE = 0.95 ‰ vs. RMSE = 0.96 ‰). This suggests that using average parameters for berry producing plants will be sufficient for a high-quality model performance. In fact, even a model parameterization with general values obtained for leaves and averaged across various species results in a relatively good performance of the model. The robust performance of the differently parameterized models is an important outcome from this work, as it suggests a general applicability of the mechanistic plant physiological stable oxygen isotope model for simulating the spatial variability of δ^18^O values in fruit. However, our analysis also shows that a careful choice of model input data that are specific for the year and month of sample collection–with varying lead times—is crucial for the performance of the model.

We found that model simulations using model parameters defined for berry producing plants (both average across berry producing plants and strawberry-specific) yielded model outputs that were slightly superior to those using at least one general parameter obtained from averaging leaf-derived literature values across species (Fig. 2). Berries, like most other fruit, are largely heterotrophic (carbon-sink) tissues, relying on sugars imported from active photosynthetic organs (i.e. leaves). These sugars carry a climate and physiologically driven leaf water δ^18^O signal^21,22,26,33,34^. This leaf water δ^18^O value that is imprinted into sugars with a fixed fractionation of + 27 ‰ can be calculated by the Craig-Gordon model if the model is amended by the parameter *f*_*xylem*_ that accounts for the dilution of the evaporatively ^18^O-enriched leaf water by the plant’s source water^35^. For the two best performing models we used *f*_*xylem*_ values that were recently determined from independent growth chamber experiments either for berry producing plants in general (0.26), or specifically for strawberry plants (0.30)^28^. These *f*_*xylem*_ values were slightly higher than mean *f*_*xylem*_ values that have been reported on average for leaves from various species in the literature (0.22)^36–40^. Higher *f*_*xylem*_ values could be the result of higher transpiration rates in berry producing plants used in agriculture than in the previously investigated plants that delivered the average *f*_*xylem*_ values of 0.22, and that included less productive wild plants with possibly lower transpiration rates.

During sugar transport from leaves via the phloem to carbon-sink tissues such as berries and during the carbohydrate synthesis in the sink tissues, some of the oxygen of the sugars exchanges with the surrounding water^41,42^. This water is ^18^O-depleted compared to leaf water^43^. Therefore, the δ^18^O values of sugars and carbohydrates in sink tissues differ from those of leaves^28,44^. In the model, the parameter p_x_p_ex_c accounts—among others—for this effect. Our analysis shows that using p_x_p_ex_c values that are specific for berries in berry producing plants improved the fit of predicted strawberry dried tissue δ^18^O values to those of reference samples. The p_x_p_ex_c value for berry bulk dried tissue that we obtained from independent growth chamber experiments (which are identical for strawberries and berries in general)^28^ and used for the best performing model simulations was higher than the mean value typically reported for cellulose of leaves (0.46 vs 0.40^45–49^). A higher p_x_p_ex_c value in berries compared to the p_x_p_ex_ value of leaf-cellulose could be the result of increased oxygen exchange in sugar with plant source water (p_ex_) during phloem transport from leaves to berries, as well as during carbohydrate synthesis in the berries. It could also indicate that berries typically contain more ^18^O-depleted source water than leaves (p_x_), which is in line with a recent observation in strawberries and raspberries^28^. Further, this pattern clearly shows that bulk dried tissue of berries compared to pure cellulose of leaves contains compounds with lower δ^18^O values than cellulose itself^50^, accounted for by a higher p_x_p_ex_c value.

For differently parameterized models, using mean air temperature of the more coarsely spatially resolved CRU dataset (0.5° grid) resulted in an improved model fit compared to the finer resolution E-OBS dataset (0.1° grid) (Fig. 2). Other than the spatial resolution, differences between the two data sources are not evident, and the E-OBS and CRU mean temperature of the sampling month of the reference samples correlated with an r^2^ of 0.98 (y = 1.04x − 0.007) for the sites used in this study. Although the best performing model used precipitation δ^18^O values from Piso.AI, which uses station coordinates and provides values for single months and years, we found model prediction skill to be generally better when using long-term monthly climatology-based precipitation δ^18^O data from the gridded OIPC datasets as input data, as opposed to Piso.AI. It is somewhat surprising that lower spatial resolution, and climatological as opposed to contemporaneous input data generally resulted in more accurate model fits than those obtained from inputs that more closely reflect conditions at any point and time. This may relate to the fact that the exact growing location and collection date of the reference samples that we used as a validation target were often only broadly defined in the sample metadata (typically only postal codes or region names, and the month of sample delivery to the lab). This may have resulted in misassignments of climate or precipitation δ^18^O values from neighboring nearby locations when using the more highly spatially and/or temporally resolved E-OBS and Piso.AI products, as opposed to the coarser products from CRU and OIPC. Also, when comparing Piso.AI to OIPC, Piso.AI generally produces a larger annual cycle than OIPC, so while picking the correct integration time can result in a better fit (see best preforming model), picking an incorrect one can result in bigger consequences for being wrong. Moreover, the differentiation between OIPC and Piso.AI is not only between data sources, but also between the timescales of integration. It may be the case that by averaging on interannual timescales, the monthly climatology precipitation δ^18^O values from OIPC more accurately replicate typical δ^18^O values of the source water that the plants use, which integrate over the timescales of the seasonal cycle due to the often long residence time of water in soil prior to plant uptake^51,52^. Both processes have the effect of smoothing out extreme δ^18^O values that may occur in any individual month. If this explanation is correct, it also implies that predictions may be improved in the future through additional validation work using new reference samples collected with more rigorous metadata.

Our model output comparison shows that using climate input variables for the growing season of a specific year substantially improved the predictive power of the model. This is exemplified by the improvement of model predictions of the average berry parameterized model using growing season specific input data (RMSE = 0.96 ‰) compared to using 11-year average (2007 to 2017) mean growing season input data (RMSE = 1.27 ‰). Critically important for an accurate application of a plant physiological model to simulate δ^18^O values of bulk dried tissue for any given growing season, is also the time span before sample collection (i.e. during which a product grew) for which climatic input data are averaged before they are entered into the model. We found that using average values for air temperature and vapor pressure from one month before sampling (which is the time during which the berry grew) is the most suitable time span for which climatic input data should be averaged for simulating the δ^18^O values in berry bulk dried tissue. The reason for improved model predictions when inter- and intra-annual variability climatic model input variable is accounted for comes from the fact that climatic drivers of leaf water and thus bulk dried tissue δ^18^O values in plants, i.e. temperature and vapor pressure, can vary substantially from year to year and within a year. As such, leaf water δ^18^O values also vary from year to year and within the growing season. This inter- and intra-annual variability in leaf water variability δ^18^O values is imprinted into the δ^18^O values of plants and can cause substantial temporal variability of plant organic δ^18^O values at a given geographic location^46,53^. The option to simulate this variability by using climatic input variables for specific years or specific parts of the growing seasons is thus a major advantage of plant physiological stable isotope models in the origin identification of plant samples compared to the conventionally used reference datasets, which can typically not be established for a specific growing season.

**Figure 4:**
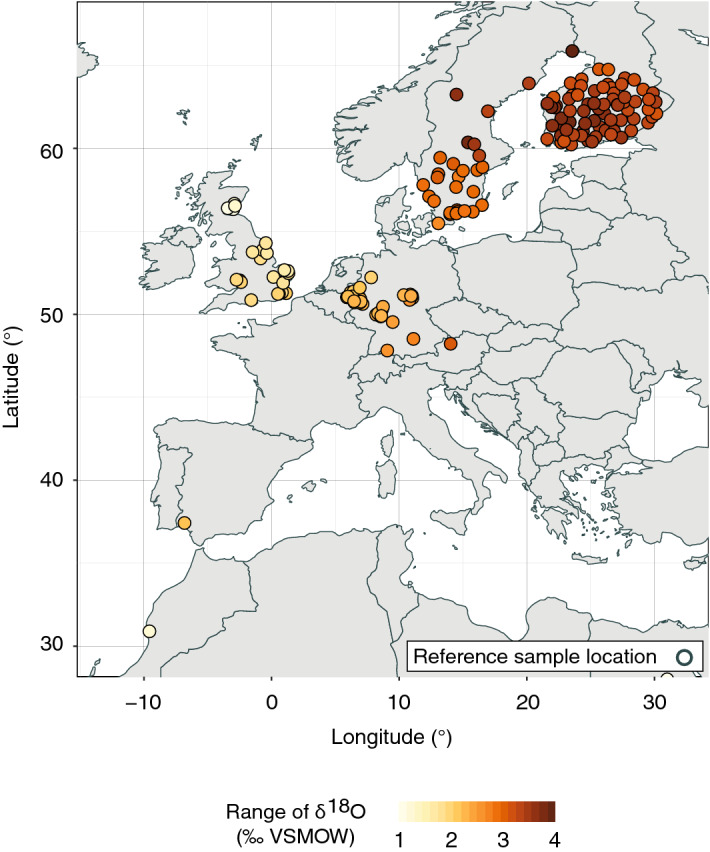
Map of Europe, showing locations where authentic reference samples were collected, and the range of predicted strawberry bulk dried tissue δ^18^O values at each location for all growing season months (May to July), and years over which the sample set was collected (2007–2017). Predictions were made using the best berry-specific model (see text). The map was created using the software R, version 3.5.3 (https://www.r-project.org/).

We demonstrate the extent of climate induced interannual variability in bulk dried tissue δ^18^O values of strawberries that can be captured by using growing season-specific model input variables for all locations from which authentic reference samples were collected for in this study (Fig. 4). For this purpose, we simulated bulk dried tissue δ^18^O values for strawberries for the three growing season months (May–July) for each of the 11 years from 2007 to 2017 (33 values per site) during which the reference samples were collected (see methods for the details of this model simulation). We then determined the maximum range of values in the resulting 11-year time series for each sampling location (Fig. 4). The data show interannual variability in bulk dried tissue δ^18^O values of strawberries of up to 4.10 ‰. Interestingly, the interannual variability in bulk dried tissue δ^18^O values of strawberries shows distinct geographical patterns with lower variability in southern and mid-range latitudes, and higher variability in northern latitudes. The apparent latitudinal pattern in site-specific predicted ranges of δ^18^O values was linear and statistically significant (r^2^ = 0.65, *p* < 0.001). These distinct geographic patterns are likely due to a higher seasonal climate variability in higher latitudes. Our analysis shows, that using growing season specific climatic input variables is increasingly important for the simulation of bulk dried tissue δ^18^O values of fruit at higher latitudes. The widely used approach of comparing suspicious samples of annually growing agricultural products to reference data, often collected over several years, cannot resolve this uncertainty, although as a compromise solution for the classic reference dataset approach, multiple reference datasets might be built up over time for different months.

Compared to climatic model input data, the temporal choice of precipitation and vapor δ^18^O data is less important for an accurate simulation of the measured values. We show that for the best two parameterized models with the best timing of climate, any combination of precipitation and vapor oxygen isotope input, predicts the measured strawberry δ^18^O bulk dried tissue values with an accuracy between RMSE values of 0.95 ‰ and 0.96 ‰, to 1.17 ‰ and 1.20 ‰, respectively (Fig. 2 g,i). Studies that have assessed the seasonal pattern of precipitation, soil and plant source water δ^18^O values, have also identified lags time between precipitation and the source water δ^18^O values of plants. It has been shown that plants use water precipitated during and, mainly for trees, prior to the growing season^51,54,55^. Our results imply that source water for strawberries integrates precipitation events before and during the berry growth, and that the model is robust to variation in precipitation and vapor δ^18^O input data between years so that possible anomalies will not strongly influence the model’s prediction power.

It is important to note that next to the quality and timing of the model input data, precise claims about the suspicious plant sample’s location of origin and picking date are required for the type of specific origin analysis that our approach facilitates. In case of using the berry-specific model input parameters, a one-month error in the metadata, would reduce the model prediction power through use of misassigned input data, resulting in a 0.33 ‰ larger RMSE. Moreover, it should also be mentioned, that the model application discussed here was developed for strawberries grown in open field conditions, rather than in greenhouses. Thus, for a successful application of the model, metadata verifying the natural growing conditions of a suspicious sample is crucial.

**Figure 5:**
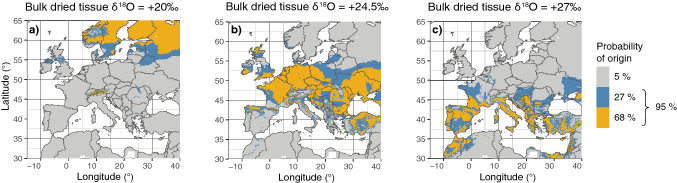
Prediction maps of three strawberry samples of unknown origin collected in July 2017. The prediction model is based on the best berry-specific model (Fig. 3) and shows the probability of origin (higher than 32% in yellow, 5% to 32% in blue, and lower than 5% grey). Bulk dried tissue δ^18^O value of sample: (**a**) + 20 ‰ (mean Finish/Swedish sample), (**b**) + 24.5 ‰ (mean German sample), and (**c**) + 27 ‰ (mean southern European sample). Prediction maps were calculated by subtracting the δ^18^O values of the bulk dried tissue of the suspected sample from the mapped results of the best berry-specific model. This resulted in a map showing the places that are predicted to have the same δ^18^O values as the sample as a value of zero. The bigger the difference shown on the map, the lower the probability of provenance of the sample. Based on the prediction error of the best berry-specific model (RMSE = 0.96 ‰), the one sigma (68%, yellow) and two sigma (95%, yellow and blue) confidence intervals around the areas showing no difference to the δ^18^O value of the suspected sample can be shown. The three maps were created using the software R, version 3.5.3 (https://www.r-project.org/).

To illustrate the power of the Craig-Gordon-derived plant physiological isotope model to predict the origin of an unknown sample for a given growing season, we produced prediction maps for δ^18^O values of bulk dried tissue from three example strawberries collected in July 2017 using the best average berry model. This type of prediction map is the main product of interest for the food forensic industry because it shows all regions of possible origin of a sample of unknown provenance. The simulations show that samples from northern Europe, central Europe, and southern Europe can be clearly distinguished (Fig. 5). The assignment maps also showed that geographic regions can be distinguished within many individual countries. For example, small parts of mid-western Germany (Cologne Area) and south-western France showed similar δ^18^O values as southern European samples (Fig. 5c).

In our study we demonstrated how plant physiological stable oxygen isotope models can produce temporally resolved, accurate, and precise region-of-origin assignments for agricultural food products. The applicability of model-based region-of-origin assignments for agricultural plant products will add several benefits for the isotope-based detection of food fraud. One major benefit is that the model is robust and does not necessarily require a species-specific parameterization. With this, the model can be applied not only to strawberries as we show as an example here but also to other agricultural plant products or regions of the world with little change in the model parameters. This guarantees that the model can be rapidly applied to constrain the geographic origin of any type of agricultural plant product, although some degree of validation work with authentic samples would still be advisable.

Another major benefit of the model is the option to simulate year- and season-specific δ^18^O values of a target plant product and to account as such for the potentially large temporal variability in such samples. This is often not possible when conventional reference isotope datasets are employed for origin validation. Finally, the modeling approach we present has the ability to identify all possible locations of provenance, without the need for spatially and temporally extensive reference samples across the entire potential region of origin. As such, it can also be used as a primary tool to efficiently acquire knowledge of all possible regions of origin of a suspicious sample, and in so doing provide specific advice about where to best collect new and/or additional reference samples.

Although predictions based on modeled δ^18^O values alone are clearly not accurate enough to define the exact growth region, they are a marked improvement and complement to using a reference sample-based approach. When combined with other techniques, such as stable isotope analyses of hydrogen, nitrogen, carbon, or sulfur, which also show distinct geographical patterns depending on underlying bedrock/mineralogy or agricultural practice and have routinely been used in origin determination^11,56^, and together with other authentication methods like e.g. proteomics or trace element analysis^10^, the possible regions of origin may be even further constrained. These techniques can also be further refined by masking regions where a given agricultural plant product cannot be or is known not to grow. Our study therefore shows that using product independent input data (climate and precipitation stable isotope values), plant physiological isotope models offer a new and powerful tool that can help to advance the use of stable isotopes to counter food fraud.

## Methods

### Independent reference samples

The authentic, independent strawberry (*Fragaria* × *ananassa*) reference samples used for model validation in this study were provided by Agroisolab GmbH (Jülich, Germany). The samples were collected either directly by the company or on their behalf through authorized sample collectors between 2007 and 2017. The primary purpose of such authentic reference samples is the direct comparison between their stable isotope compositions (oxygen, hydrogen, carbon, nitrogen, or sulfur) to those of samples of suspect origin. Accompanying metadata for each reference sample included information about the geographic origin as community name, postal code, or location coordinates, and information about the month and year the strawberry sample was picked. In total, we used δ^18^O values from 154 reference samples. Most samples were collected in the UK, Germany, Sweden, and Finland (Fig. 1). All reference samples were grown on open strawberry-fields rather than artificial greenhouse conditions. All berry samples were collected from cultivated, non-endangered plant species (“garden strawberry”), and the research conducted complies with all relevant institutional, the corresponding national, and also international guidelines and legislation.

After collection in the field, samples were stored in airtight containers and shipped directly to Agroisolab, where they were stored frozen prior to analysis. In order to analyze the oxygen stable isotope composition of the organic strawberry tissue, the lipids were solvent-extracted with dichloromethane for a at least 4 h, using a Soxhlet extractor. The remaining samples were dried and milled to a fine powder. 1.5 mg of the powder was weighed into silver capsules. The silver capsules were equilibrated for at least 12 h in a desiccator with a fixed relative humidity of 11.3%. After a further vacuum drying the samples were measured via high-temperature furnace (Hekatech, Wegberg, Germany) in combination with an Isotope-ratio mass spectrometer (IRMS) Horizon (NU Instruments, Wrexham, UK). The pyrolysis temperature was 1530 °C and the pyrolysis tube consisted of covalent-bound SiC (Agroisolab patented). The reproducibility of the measurement was better than 0.6 ‰.

### Oxygen isotope model calculation

Plant physiological stable isotope models simulate the oxygen isotopic composition of leaf water or organic compounds synthesized therein as δ^18^O values in per mil (‰), where δ^18^O = (^18^O/^16^O)_sample_/(^18^O/^16^O)_VSMOW_ − 1, and VSMOW is Vienna Standard Mean Ocean Water as defined by the VSMOW-Standard Light Antarctic Precipitation (SLAP) scale. The Craig-Gordon model^57^, which was developed to mathematically describe the isotopic enrichment of standing water bodies during evaporation and later modified for plants, is the basis for modelling plant water δ^18^O values^23,58^. Plant source water is the baseline for the model, which is the precipitation-derived soil water that plants take up through their roots without isotope fractionation^51,59,60^. The ^18^O enrichment of water within leaves is described by the following equation (Eq. 1)^36,61^:

1 $$ \Delta^{18} {\text{O}}_{{\text{e leaf}}} = \left( {1 +\upvarepsilon ^{ + } } \right)\left[ {\left( {1 + {\upvarepsilon }_{{\text{k}}} } \right)\left( {1 - {\text{e}}_{{\text{a}}} /{\text{e}}_{{\text{i}}} } \right) + {\text{e}}_{{\text{a}}} /{\text{e}}_{{\text{i}}} (1 + \Delta^{18} {\text{O}}_{{{\text{Vapor}}}} )} \right]{-}1 $$

where Δ^18^O_e_leaf_ is the oxygen isotopic enrichment above source of water at the evaporative site in leaves, ε^+^ is the equilibrium fractionation between liquid water and water vapor, ε_k_ is the kinetic fractionation associated with the diffusion through the stomata and the boundary layer. e_a_/e_i_ is the ratio of ambient vapor pressure in the atmosphere to intercellular vapor pressure in the leaf. Δ^18^O_Vapor_ is the isotopic composition of the ambient vapor above source water, which in this study is assumed to be in equilibrium with the source water (Δ^18^O_V_ = − ε^+^)^62,63^. This assumption can be used, if the atmosphere is well mixed, and plants’ source water derives from recent precipitation events. For crops, growing in the temperate climate of the mid latitudes this is usually the case, especially over the long time periods (several weeks) over which strawberries grow. If such a model is applied in other climatic zones (e.g. tropics), this assumption should, however, be reevaluated^64^. The equilibrium fractionation factor (ε^+^)^65,66^ and kinetic fractionation factor (ε_k_)^67^ can be calculated with the following equations (Eqs. 2 and 3):

2 $$\upvarepsilon ^{ + } = \left[ {\exp \left( {\frac{1.137}{{\left( {273 + T} \right)^{2} }}*10^{3} - \frac{0.4156}{{273 - T}} - 2.0667*10^{ - 3} } \right) - 1} \right]*1000 $$

where T is the leaf temperature in degrees Celsius. In our calculations, leaf temperature was set to 90% of the monthly mean air temperature, which describes a realistic leaf-energy balance scenario for well-watered crops^68,69^, and also yielded the best model performance with respect to the reference data. As leaf to air temperature differences have a strong influence on leaf water δ^18^O values, this assumption needs to be independently tested in future applications. For example, changing leaf temperature from 20 °C to 22 °C at a constant air temperature of 20 °C and a source water δ^18^O value of -10 ‰ will affect leaf water δ^18^O values by + 1.4 ‰.

3 $$\upvarepsilon _{{\text{k}}} = \frac{{28{\text{r}}_{{\text{s}}} + 19{\text{r}}_{{\text{b}}} }}{{{\text{r}}_{{\text{s}}} + {\text{r}}_{{\text{b}}} }} $$

where r_s_ is the stomatal resistance and r_b_ is the boundary layer resistance in m^2^s/mmol, which is the inverse of the stomatal and boundary layer conductance. For our model calculations, we consistently used stomatal conductance values of 0.4 mol/m^2^s, stomatal resistance values of 1 m^2^s/mol^70^.

The Craig-Gordon model predicted leaf water values are often enriched in ^18^O relative to measured bulk leaf water δ^18^O values^26,27^. This is because the model describes the δ^18^O values of water at the site of evaporation while measurements typically give bulk leaf water δ^18^O values^36,71^. The two-pool modification to the Craig-Gordon model corrects for this effect by separating bulk leaf water into a pool of evaporatively enriched water at the site of evaporation (δ^18^O_e_leaf_ derived from the Craig-Gordon model, Eq. 1) and a pool of unenriched plant source water (δ^18^O_source water_)^25^. δ^18^O_e_leaf_ is calculated as follows:

4 $$\updelta ^{18} {\text{O}}_{{{\text{e}}\_{\text{leaf}}}} = \, (\Delta^{18} {\text{O}}_{{{\text{e}}\_{\text{leaf}}}} +\updelta ^{18} {\text{O}}_{{\text{source water}}} ) \, + \, (\Delta^{18} {\text{O}}_{{{\text{e}}\_{\text{leaf}}}} * \,\updelta ^{18} {\text{O}}_{{\text{source water}}} )/1000) $$

In the two-pool modified Craig-Gordon model (Eq. 5), the proportion of unenriched source water is described as *f*_*xylem*_^36^_._

5 $$\updelta ^{18} {\text{O}}_{{\text{leaf water}}} = \, \left( {1 \, {-}f_{xylem} } \right) \, * \,\updelta ^{18} {\text{O}}_{{{\text{e}}\_{\text{leaf}}}} + \, \left( {f_{xylem} * \,\updelta ^{18} {\text{O}}_{{\text{source water}}} } \right) $$

Values for *f*_*xylem*_ in leaf water generally range from 0.10 to 0.33^36–40^ but higher values have also been observed^72^. For strawberry plants, leaf water *f*_*xylem*_ values were recently shown to vary between 0.24 and 0.34^28^.

Organic molecules in leaves generally reflect the δ^18^O values of the bulk leaf water plus additional isotopic effects occurring during the assimilation of carbohydrates and post-photosynthetic processes^21,22,34^. The fractionations occur when carbonyl-group oxygen exchanges with leaf tissue water during the primary assimilation of carbohydrates (trioses and hexoses)^42^. This process causes ^18^O enrichment, described as ε_wc_^42^, and has been determined to be ~  + 27 ‰^21,22,73^.

During the synthesis of cellulose from primary assimilates, sucrose molecules are broken down to glucose and re-joined, allowing some of the carbonyl group oxygen to further exchange with water in the developing cell. The isotopic fractionation (ε_wc_) during this process is assumed to be the same as in the carbonyl oxygen exchange during primary carbohydrate assimilation (~ + 27 ‰)^41,42^. During the formation of cellulose, the δ^18^O values of the primary assimilates are thus partially modified by the water in the developing cell^33^. Equation (6) describes this process^34^

6 $$\updelta ^{18} {\text{O}}_{{{\text{cellulose}}}} = {\text{ p}}_{{\text{x}}} {\text{p}}_{{{\text{ex}}}} *\left( {\updelta ^{18} {\text{O}}_{{\text{source water}}} + \,\upvarepsilon _{{{\text{wc}}}} } \right) \, + \, \left( {1 \, - {\text{p}}_{{\text{x}}} {\text{p}}_{{{\text{ex}}}} } \right) \, * \, \left( {\updelta ^{18} {\text{O}}_{{\text{leaf water}}} + \,\upvarepsilon _{{{\text{wc}}}} } \right) $$

where δ^18^O_cellulose_ is the oxygen isotopic composition of cellulose, p_ex_ is the fraction of carbonyl oxygen in cellulose that exchanges with the medium water during synthesis, and p_x_ is the proportion of unenriched source water in the bulk water of the cell where cellulose is synthesized^33^. Bulk water in developing cells where cellulose is synthesized, i.e. in the leaf growth-and-differentiation zone, has been found to primarily reflect the isotope composition of source water^43^. Therefore, p_x_ in Eq. 6 is likely larger than *f*_*xylem*_ in Eq. (5). For practical reasons, the parameters p_x_ or p_ex_ are typically not determined individually, but as the combined parameter p_x_p_ex_^45^. For cellulose in leaves of grasses, crops, and trees p_x_p_ex_ has been found to range from 0.25 to 0.54^45–49^.

In this study as in many applied examples where plant δ^18^O values are used for origin analysis we attempt to simulate the δ^18^O values of dried bulk tissue. Bulk dried plant tissue (δ^18^O_bulk_) contains in addition to carbohydrates compounds such as lignin, lipids, and proteins, which can be ^18^O-depleted compared to carbohydrates^50^. Since this needs to be accounted for in the model, we included the parameter c into the model. As p_x_p_ex_ and c cannot be determined separately they are used as a combined model parameter in our approach p_x_p_ex_c:

7 $$\updelta ^{18} {\text{O}}_{{{\text{bulk}}}} = {\text{ p}}_{{\text{x}}} {\text{p}}_{{{\text{ex}}}} {\text{c}}*\left( {\updelta ^{18} {\text{O}}_{{\text{source water}}} + \,\upvarepsilon _{{{\text{wc}}}} } \right) \, + \, \left( {1 - {\text{ p}}_{{\text{x}}} {\text{p}}_{{{\text{ex}}}} {\text{c}}} \right) \, * \, \left( {\updelta ^{18} {\text{O}}_{{\text{leaf water}}} + \,\upvarepsilon _{{{\text{wc}}}} } \right) $$

Bulk dried tissue δ^18^O values of strawberries in Cueni et al. (in review) did not differ statistically from pure cellulose δ^18^O values in strawberries. Consequently, p_x_p_ex_ and p_x_p_ex_c are identical for strawberries and ranges from 0.41 to 0.51. This approach allows the calculation of bulk dried tissue δ^18^O values without the knowledge of cellulose δ^18^O values, which is the case for the data set used in this study, and contrasts to the approach by Barbour & Farquhar (2000), where bulk dried tissue δ^18^O values are assessed by an offset (ε_cp_) to the cellulose δ^18^O values.

### Model parameter selection

To find the best values of the key model parameters for the prediction of strawberry bulk dried tissue δ^18^O values, we used different combinations of the values for the parameters. Specifically, we compared average parameter values from the literature that were derived from leaves and parameter values that were specifically derived for berries (Cueni et al. in review) to test if a leaf-level parameterization of the model is sufficient or if a berry-specific parameterization is necessary for producing satisfying model prediction. These values were either (i) *f*_*xylem*_ and p_x_p_ex_ values reported in literature for leaf water and cellulose from various species that were averaged, (ii) values averaged for leaves (*f*_*xylem*_) and berries (p_x_p_ex_c) of berry producing plants, or (iii) values for leaves (*f*_*xylem*_) and berries (p_x_p_ex_c) specifically obtained for strawberry plants. For the general leaf-derived parameter values we used mean literature values originally obtained for leaf water and leaf cellulose δ^18^O for different species and averaged these values (0.22 for *f*_*xylem*_^36–40^ and 0.40 for p_x_p_ex_^45–49^) (Table 1). For berries (average of the values of raspberries and strawberries) the mean leaf-derived *f*_*xylem*_ value was 0.26 and the value for p_x_p_ex_c was determined to be 0.46 (Table 1) (data derived from Cueni et al. in review). For strawberry plants, the leaf-derived *f*_*xylem*_ value we used was 0.30, and the value for bulk dried tissue (p_x_p_ex_c) was determined to be 0.46 (Table 1) (data derived from Cueni et al. in review). Since the p_x_p_ex_c values of different berry species did not differ, this resulted in a total of six different model input parameter combinations.

**Table 1:** Values of the model parameters (*f*_*xylem*_ and p_x_p_ex/_p_x_p_ex_c) used for the simulations of strawberry bulk dried tissue δ^18^O values.

	*f*_*xylem*_	p_x_p_ex/_p_x_p_ex_c
General	0.22	0.40
Berry	0.26	0.46
Strawberry	0.30	0.46

### Environmental model input data selection

In order to apply the strawberry parametrized bulk dried tissue oxygen model on a spatial scale, spatially gridded climate and precipitation isotope data layers were used as model inputs. The accurate simulation of geographically distinct δ^18^O values, however, requires the use of the most appropriate and best available input variables. We therefore tested the importance of the temporal averaging and lead time of the input data relative to the picking date of the berry. We defined these collectively as the “integration time” of the input data. Climate of the growing season^46,53^, and precipitation δ^18^O values of rain-events prior and during the growing season^51,54,55^ have been shown to shape plant tissue water and organic compound δ^18^O values. The major objective of our study was thus a careful evaluation of the most appropriate type and integration time of model input variables needed for this kind of model simulation. Moreover, to find the best data source provided, we also used several different spatial climate and precipitation isotope datasets in our evaluations (Table 2).

**Table 2:** (a) Table showing the different climatic (air temperature and vapor pressure) and isotope (precipitation and vapor δ^18^O) data products, (b) as well as the different integration times and names used in the study used to simulate strawberry bulk dried tissue δ^18^O values.

(a) Data products	
Input variable	Data product sources
Climate (Air temperature, vapor pressure/ RH)	CRU, E-OBS
δ^18^O-precipitation	OIPC, Piso.AI
δ^18^O-vapor	OIPC, Piso.AI

(b) Integration times	
Name	Explanation
At month of sampling	Value of the variable from the month of sample collection
One month before sampling	Value of the variable from one month prior to sample collection
Two months before sampling	Value of the variable from two months prior to sample collection
Three months before sampling	Value of the variable from three months prior to sample collection
Four months before sampling	Value of the variable from four months prior to sample collection
Mean three months before sampling	Average value of the variable from the three months prior to sample collection (precipitation and vapor d^18^O values are amount-weighted using CRU precipitation totals)
11 year May to July mean	Average value of the variable from the growing season (May to July) averaged over the 11-year period from which samples used in this study were collected
Yearly May to July mean	Average value of the variable from the growing season (May to July) of sample collection

Two precipitation isotope data products were compared (Table 2): (1) The mean monthly precipitation δ^18^O grids by Bowen (2020), which are updated versions of the grids produced by Bowen and Revenaugh (2003) and Bowen et al. (2005) (Online Isotopes in Precipitation Calculator, OIPC Version 3.2). They provide global grids of monthly long-term mean precipitation isotope values. The resolution of these global grids is 5’. (2) Precipitation isotope predictions from Piso.AI (Version 1.01)^32^. This source provides values for individual months and years based on station coordinates^32^. Both data sets were on the one hand used for the precipitation δ^18^O input data of the model, and also to extrapolate the vapor δ^18^O values from sets (see model description above), which we treated as two individual, independent input data sets.

For the climatic drivers of the model (air temperature and vapor pressure), we used the gridded data products from the Climatic Research Unit (CRU) (TS Version 4.04)^29^ and the E-OBS gridded dataset by the European Climate Assessment & Dataset (Version 22.0e)^30^ (Table 2). The CRU dataset provided global gridded monthly mean air temperature, and mean vapor pressure with a resolution of 0.5°. The E-OBS dataset included European daily mean air temperature and relative humidity gridded data, with a resolution of 0.1 arc-degrees. We calculated monthly mean air temperature and relative humidity grid layers, on the basis of these daily mean air temperature and relative humidity grids, respectively.

Fruit tissue formation takes place over a period of several weeks leading up to picking date^46,53^. This results in a lead time between the date that best represents the mean climate conditions, and source water and vapor stable isotope signal influencing the isotope signal during tissue formation, and the picking date. As integration time of the input data, we therefore investigated lead times of 1, 2, 3, and 4 months, as well as the three months leading up to the picking date (Table 2). Moreover we also used more general European strawberry growing season averages^76^, independent of either the sampling month (yearly May to July mean) or the sampling year (2007 to 2017 May to July mean) (Table 2). Precipitation isotope data means were calculated as amount-weighted averages using CRU mean monthly precipitation data. This means that the long term mean precipitation δ^18^O values taken from OIPC were weighted by yearly specific CRU monthly precipitation totals for the case of the three months or growing season averages for individual years, and by average monthly precipitation totals (May, June, and July) from 2007 to 2017 for the long-term growing season calculation. The same assessment was also made using precipitation values from Piso.AI.

### Validation of model with reference samples

Using the plant physiological model described above, we calculated the strawberry bulk dried tissue δ^18^O values for the location and the growing time of each authentic reference sample. For the model input data, we tested variable combinations using each of the eight integration times described in Table 2, along with all combinations of the data sources outlined in Table 2. This resulted in a total of 65,536 combinations of input variables per model parameter combination (*f*_*xylem*_ and p_x_p_ex_/p_x_p_ex_c, Table 1), yielding model results to be evaluated against the measured reference samples. Our approach can be described with the following equation:

8 $$\updelta ^{18} {\text{O}}\,{\text{plant }} = f\left( {{\text{air}}\,{\text{temp}}\left( {\text{s,t}} \right),{\text{ relative}}\,{\text{humidity}}\left( {\text{s,t}} \right), \updelta ^{18} {\text{O}}\,{\text{precip}}.\left( {\text{s,t}} \right),\updelta ^{18} {\text{O}}\,{\text{vapor }}\left( {\text{s,t}} \right)} \right) $$

where δ^18^O plant is the simulated δ^18^O value of the strawberry, s is the data product for the specified input variable (Table 2), and t is the integration time of the specified variable (Table 2).

For the crucial model parameters *f*_*xylem*_ and p_x_p_ex_/p_x_p_ex_c we on the one hand used the values proposed for leaves by literature (for p_x_p_ex_), and on the other hand average of the values of raspberries and strawberries and strawberry-specific values determined from Cueni et al. (in review) (for p_x_p_ex_c). In all calculations an ε_wc_ value of + 27 ‰ was used. To calculate mean monthly relative humidity values from the provided CRU vapor pressure data, site specific elevation was extracted from the ETOPO1 digital elevation model^77^, and used to calculate the approximate atmospheric pressure. These values were then used in combination with air temperature to calculate the saturation vapor pressure after Buck (1981), in order to assess relative humidity (relative humidity = vapor pressure/saturation vapor pressure). The R-script of the model is available on “figshare”, find the URL in the data availability statement.

### Statistical analyses

Statistical analyses were done using the statistical package R version 3.5.3^79^. The relationships of the range δ^18^O values observed with latitude, and between CRU and E-OBS mean air temperature were compared with a linear regression model, and with an alpha level that was set to α = 0.05. The results of the 65,536 models for each of the six physiological parameter combinations were compared with the measured δ^18^O bulk dried tissue values of the authentic reference samples (n = 154) by calculation of the root mean squared error (RMSE).

### Calculation of prediction maps

Prediction maps showing the regions of possible origin of a sample with unknown provenance are the product that is of interest in the food forensic industry. We calculated the prediction maps shown in Fig. 5 for three example δ^18^O values of strawberries collected in July 2017: (i) + 20 ‰ representing a mean Finish/Swedish sample, (ii) + 24.5 ‰ representing a mean German sample, and (iii) + 27 ‰ representing a mean southern European sample.

The prediction maps were calculated in a two-step approach. First, we calculated a map of the expected strawberry bulk dried tissue δ^18^O values of berries grown in July 2017. For this we used the average berry model input parameters (*f*_*xylem*_ and p_x_p_ex_c, Table 1), and the best fitting model input data and integration time combination, which we assessed beforehand (Fig. 2). We thus used CRU mean air temperature and vapor pressure from June 2017, precipitation δ^18^O values from OIPC as an average from April, May and June, and vapor δ^18^O values calculated from OIPC precipitation δ^18^O values from April. Since using spatial maps as model input data, this calculation resulted in a mapped model result. In a second step, we calculated the prediction maps. For this we first subtracted the δ^18^O value of the bulk dried tissue of the sample strawberry from the mapped result of the best berry-specific model. This was done for each pixel value of the map. This resulted in a map showing the difference of the sample δ^18^O value and the predicted map δ^18^O value for each pixel of the map. The places (pixels) that are predicted to have the same δ^18^O value as the sample strawberry thus are represented by a value of zero. Based on the prediction error of the best berry-specific model (RMSE = 0.96 ‰), the one sigma (68%) and two sigma (95%) confidence intervals around the areas showing no difference to the δ^18^O value of the suspected sample could be assessed. This means that the bigger the difference between the simulated δ^18^O value and the sample value, the lower the probability of provenance of the sample. In other words, a difference between the sample’s δ^18^O value and the predicted δ^18^O value of 0 ‰ to ± 0.96 ‰ equals a possible provenance of at least 68% (one sigma), and a difference between ± 0.96 ‰ and ± 1.92 ‰ reflects a possible provenance between 68 and 27% (two sigma). Regions on the map with bigger differences than ± 1.92 ‰ represent regions of possible provenance, lower than 5% (bigger than two sigma).

## Supplementary Information

Supplementary Legends.

Dataset S1.

Dataset S2.

Dataset S3.

Dataset S4.

Dataset S5.

Dataset S6.

## Data Availability

The data that support the findings of this study are available as supplementary information of this publication and on “figshare” (dx.doi.org/10.6084/m9.figshare.15049260).
