# Supplementary material for: Using plant physiological stable oxygen isotope models to counter food fraud
Source: Sci Rep. 2021 Aug 27;11:17314. doi: 10.1038/s41598-021-96722-9 (PMC8397719; doi:10.1038/s41598-021-96722-9)
Supplement: Supplementary file 1 — Supplementary Legends. [file 41598_2021_96722_MOESM1_ESM.pdf]

Using plant physiological stable oxygen isotope models to counter food fraud - Supplementary Information

Florian Cueni*[1,2], Daniel B. Nelson[1], Markus Boner[2], Ansgar Kahmen[1]

[1]University of Basel, Department of Environmental Sciences – Botany, Schönbeinstrasse 6, 4056 Basel, Switzerland

[2]Agroisolab GmbH, Professor-Rehm-Strasse 6, 52428 Jülich, Germany

* Corresponding author: f.cueni.plantphys@gmail.com

Supplementary Information dataset S1: CSV-file with all model results of the input data comparison using model input parameter values of for f_{xylem} and p_xp_{ex} as averages of values reported for leaf water and cellulose, respectively, by literature (Fig. 2).

Supplementary Information dataset S2: CSV-file with all model results of the input data comparison using model input parameter values averaged for leaves of berry producing plants for f_{xylem} and average leaf literature values for p_xp_{ex} (Fig. 2).

Supplementary Information dataset S3: CSV-file with all model results of the input data comparison using model input parameter values averaged for leaves of strawberry plants for f_{xylem} and average leaf literature values for p_xp_{ex} (Fig. 2).

Supplementary Information dataset S4: CSV-file with all model results of the input data comparison using model input parameter values averaged for leaves as reported by literature for f_{xylem} and values specifically obtained for berries of berry producing plants or strawberries for $p_xp_{ex}c$ (Fig. 2).

Supplementary Information dataset S5: CSV-file with all model results of the input data comparison using model input parameter values averaged for leaves of berry producing plants for f_{xylem} and values specifically obtained for berries of berry producing plants or strawberries for $p_xp_{ex}c$ (Fig. 2).

Supplementary Information dataset S6: CSV-file with all model results of the input data comparison using model input parameter values averaged for leaves of strawberry plants for f_{xylem} and values specifically obtained for berries of berry producing plants or strawberries for $p_xp_{ex}c$ (Fig. 2).
